# Setting priorities in outpatient cardiovascular care to guarantee equitable access: the case of Tuscany region

**DOI:** 10.1007/s43999-024-00047-9

**Published:** 2024-08-07

**Authors:** Vera Benedetto, Erica De Vita, Sabina Nuti

**Affiliations:** Interdisciplinary Research Center “Health Science”, Scuola Superiore Sant’Anna, Via Cardinale Maffi 27, Pisa, Italy

**Keywords:** Equity, Unwarranted variation, Cardiovascular care, Waiting times, Visit rates

## Abstract

Universal healthcare systems orient their actions towards promoting, restoring, and improving public health with a particular focus on the need to guarantee equitable access to care. Unwarranted variation in healthcare delivery poses significant challenges to health systems globally, impacting quality of care, financial sustainability, and equity of access. It is therefore important to assist healthcare management in measuring unwarranted variation in order to prioritise intervention strategies to ensure continuity of care and equity. Through an investigation of geographical variation in visit rates and waiting times, the study identifies vulnerable health districts which need priority interventions for patients with cardiovascular disease in the Tuscany region (Italy). Furthermore, a benchmarking-based method for identifying a quantitative estimate of the supply gap to be reduced is proposed. Results illustrate variation in visit rates and waiting times across local health districts in 2021, with some districts experiencing substantially lower rates and longer waiting times compared to the regional median. To address this gap, two targeted interventions aimed at increasing visit volumes and reduced waiting times through advanced training activities, technology integration, and multidisciplinary collaborations are presented. This study contributes to the topic of unwarranted variation by highlighting the necessity of tailored interventions to address diverse healthcare challenges across heterogeneous geographical areas. As healthcare systems globally navigate evolving complexities, the findings and tools presented here offer valuable guidance for policymakers and managers, aiming towards more equitable, efficient, and responsive healthcare services.

**Supplementary Information:**

The online version contains supplementary material available at 10.1007/s43999-024-00047-9.

## Background

Unwarranted variation is one of the most problematic issues in health care systems, impacting quality of care, financial sustainability and equity of access. Indeed, the presence of variation in healthcare delivery, not driven by patient preferences or health requirements, but influenced by factors such as resource allocation, the number of healthcare professionals in a given geographic area, their competences, and the presence or absence of shared clinical protocols [1, 2], represents a considerable challenge for health care systems. This variation not only constitutes a source of potential financial wasting but also increases the risk of supply gaps that may adversely impact the health and outcomes of the served population.

This theme has been studied for many years now, starting from the work of Wennberg and subsequently by many other authors [3–9]. With increasing awareness of this issue, institutional bodies and healthcare organisations have generated reports to aid policymakers and health managers. These reports aim to enhance public knowledge and suggest potential interventions, particularly within the hospital sector [10, 11]. For example, the King’ Fund [10] has identified common factors contributing to variations in health care, e.g. differences in patients’ needs arising from demographic structure and socio-economic conditions, clinical decisions that may vary among healthcare providers, lack of standardised guidelines or protocols for certain medical interventions and inadequate data collection and analysis on variations in health care delivery. Hence, to reduce unwarranted variation, several suggestions are proposed in the document, such as prioritising variations and causes that have the most significant impact on equity, systematic and routine collection, analysis, and publication of data on variations in health care, and encouraging shared decision-making to establish the right level of variation based on patients’ assessments of needs and risk aversion.

The issue of unwarranted variation is even more crucial when it is intertwined with that of waiting times, which represents a real problem for policy makers and health managers as it strongly impacts on consensus and on the evaluation of health services by citizens [12–14].

Ensuring timely visits is a critical component of healthcare service quality, because it can influence the effectiveness of care [15]. The challenge of prolonged waiting times entwined with unwarranted variation, has been addressed in the literature through various studies.

Given that the Italian National Health Care System (NHS) provides universal coverage for comprehensive and essential health services through general taxation, decisions regarding resource allocation in terms of supply have the potential to make a difference in diminishing unwarranted variation between geographical areas. Additionally, such choices can contribute to the reduction of waiting times, a common symptom of a supply gap, particularly when coupled with a low rate of services provided per inhabitant. Thus, managers and policymakers should adopt tools for measuring both unwarranted geographic variation in the provision of care and waiting times, identifying areas of missing health services, and implementing effective strategies to improve equity and care.

Some studies have explored the intersection of waiting times and rates of visit delivered across various services, e.g. radiology [5]. In this context, Nuti and Vaineri [5] introduced a conceptual framework for mapping geographical variations in both waiting times and service outcomes onto a single matrix. The tool comprises a two-dimensional matrix, with the x-axis representing the utilisation rate of a specific health service (volumes per inhabitants), and the y-axis depicting the waiting times for service delivery. This graphical visualisation technique provides managers with a targeted tool for evaluating a potentially critical supply of a certain service, identifying potential solutions, and implementing interventions.

Tuscany is a large region in central Italy that, at the local level, is geographically based on local health authorities (LHAs) which are in charge of organising and providing health services, from primary care to prevention services. To strengthen operational capacity and reduce variation, in 2015, the Tuscany Region initiated a process of concentration over the years by merging the 12 local health authorities into three large ones (Regional Law n. 84, 2015), Northwest LHA, Centre LHA, and Southeast LHA, that are in charge of 26 local health districts. The objective was to empower Local Health Authorities (LHAs) to address and reduce avoidable geographical variations among districts, thereby expressing enhanced allocative strategies, i.e. facilitating resources mobility among districts, which are the optimal geographical area for assessing the health and social needs of communities and for organising and delivering services within integrated health, socio-health, and social territorial networks.

## Focus and aim

With these premises, the study presents the case of the outpatient cardiological visits delivered in Tuscany during the period 2019–2022. These visits are the most frequent and numerous among the ambulatory services provided by Tuscany and generally by the Italian NHS [16]. During the last decades, the increase in the incidence of cardiovascular diseases and ageing of the population with associated comorbidity or multimorbidity [17–19], along with advances in treatment and preventive measures [20, 21], was accompanied by a greater number of patients needing medical attention from cardiological services [22].

Moreover, the Covid-19 pandemic has placed health systems worldwide under unprecedented pressure, unleashing pre-existing troubles. The pandemic led Italian regions and providers to prioritise urgent treatments reducing overall outpatient services on one side, and on the other discouraging patients from attending appointments for fear of contagion. With the rapid spread of the Covid-19 across Italy, the ability to address and respond to non-communicable diseases (NCDs) has been negatively impacted [23].

In this context, the paper aims to:

- Examine the yearly variation from 2019 to 2021 of cardiology visits to assess the responsiveness to the health needs of the population across the 26 health districts in the Tuscany region (Italy) during this period.
- Intersecting visit rates and waiting times by applying the matrix proposed by Nuti & Vainieri [5], to identify those districts with a higher lack of offer, thus with the highest demand for management intervention.
- Identify geographical areas with the greatest disparities in visit delivery and extended waiting times, and explore whether, despite the challenging circumstances linked to the Covid-19 pandemic, a significant gap persists.
- Prioritise and quantify actions in the most neglected districts by identifying required interventions based on the volume of visits needed in districts with both low visit rates and extended waiting times, in comparison to the regional median. Although a clinical standard rate does not exist for the cardiology visit rates, this indicative number can be a point of reference for the Region and for the Local Health Authorities.

### Matrix for waiting times and visits rates

**Fig. 1:**
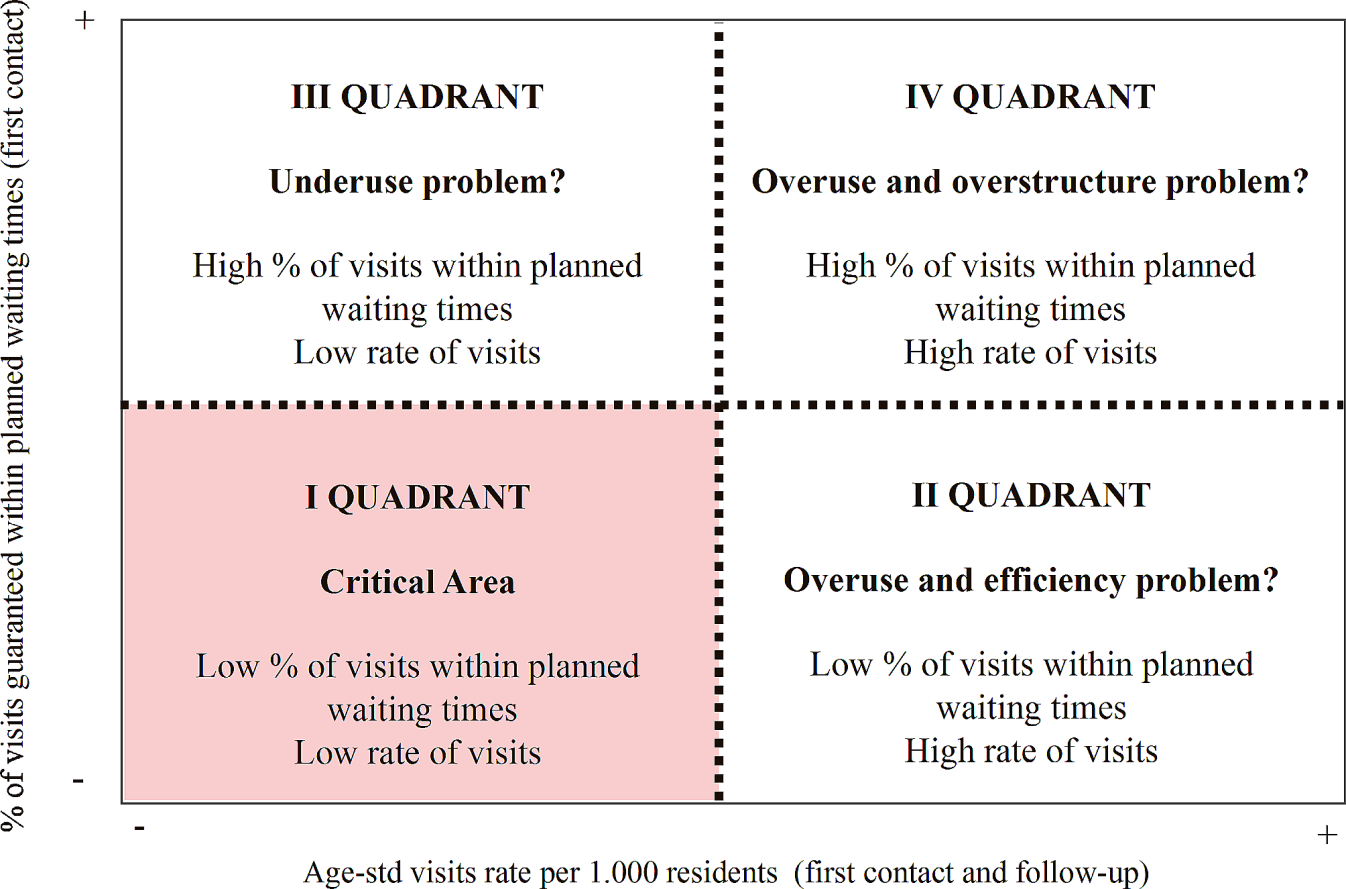
Framework for waiting times and age-standardised cardiology visits rate per 1000 residents. First contact and follow-up visits have been considered together

The matrix proposed by Nuti and Vaineri [5] is a valuable logical tool for identifying geographical variation and ultimately helping decision makers define the priorities of intervention. More specifically, the logical matrix proposed in the study involves jointly analysing waiting times and volumes of diagnostic imaging services. For our study, we have adapted the matrix by considering waiting times on the y-axis and the sum of first contact and follow-up cardiology visits rates on the y-axis. Then, we drew a line in correspondence of the median value of the moving average of both indicators for the years 2019–2021 (henceforth median) obtaining four quadrants (Fig. 1). This approach was employed to enhance comparability and yield a more robust measure of tendency across the considered years as well as to reduce sensitivity to extreme values and serves to mitigate the impact of outliers within our analytical framework. Moreover, each local health district has been identified with a colour code to visualise the management practices and resource allocation within the local health authorities – Northwest (red colour), Southeast (green colour), and Centre (blue colour). This visualisation allows to point out those health districts that may need more support regarding managerial interventions.

Each quadrant requires different and specific interventions; hence strategies differ among all quadrants. If districts are in the fourth quadrant, i.e., short waiting times and high cardiology visits rates per 1000 inhabitants, managers and clinicians need to inquire whether there is a risk of delivering inappropriate services and/or having excessive resources at their disposal, leading to overstaffing and oversupply. For districts located in the third quadrant, managers and clinicians should examine whether healthcare systems may be witnessing an underutilization issue: this situation could lead to a reduction in service supply and a drop in quality if patients decide to be treated elsewhere. If districts are in the second quadrant, managers and clinicians should check whether the long waiting times and high rates of cardiology may be due to inappropriateness in the prescription or production efficiency issues. However, a very different situation exists if health districts belong to the first quadrant - i.e., the most critical area, indicated in red in *Fig. 1*. Indeed, long waiting times and low rates of cardiology visits per 1000 inhabitants may indicate an inadequate capacity to provide essential services, i.e. a lack of offer. Patients in these areas face a two-fold problem: low rates of cardiology first contact and follow-up visits, which could be a symptom of supply gap, and long waiting times. This results in patients experiencing extended waiting times compared to other local health districts when seeking services. In this quadrant, responsibilities appear to weigh more heavily on management compared to other quadrants, as it is more likely that these areas suffer from a resource allocation issue rather than a prescriptive problem. Additionally, organising and sizing the supply of services is a specific responsibility of management.

## Methods

### Data

We conducted a retrospective study using outpatient administrative data of the region of Tuscany, Italy, from 2019 to 2022. The analysis included all patients, regardless of age, linked to first contact and follow-up cardiology visits regional codes (Decree n. 16,269, October 14, 2020, Tuscany Region): 1089 (first contact visit); P009 (consultation first contact visit); 1192 (follow-up visit); P010 (consultation follow-up visit); 2634 (follow-up video consultation); 2657 (follow-up paediatric video consultation). For each provision, administrative databases in Tuscany enabled us to identify the local health unit where the patient lives, the facility that provides care (name of the facility and if public or private), the pathway (if it is a first contact or a follow-up), and the time when the visit was prescribed. Records linked to patients that do not reside in Tuscany and patients accessing the emergency department have been excluded to faithfully depict the ability of the region to provide non-emergency care to its citizens. In addition, the Regional Health Information System office assigned a unique identifier to each patient, which does not disclose the identity of patients or other sensitive data, thus ensuring compliance with Italian privacy laws. Given that we used administrative data, an ethics committee approval was unnecessary.

### Indicators

The study includes the following indicators relevant to assessing the health system’s management response to unwarranted geographical variation based on the Tuscan Performance Evaluation System (PES):

- Age-standardised cardiology visits rate. The age-standardised cardiology visits rate – henceforth cardiology visits rates – computes, for the years 2019–2022, the percentage of cardiology visits for 1,000 residents for each local health district of the Tuscany region. This indicator has been split into first-contact visits, repeated first-contact visits, and follow-up visits.
- Percentage of first-contact cardiology visits guaranteed within Tuscany’s Regional waiting list plan (Regional Waiting List Regulation Plan – Ordinance of the Regional Council of Tuscany 604/2019) time frame (first visits) – henceforth waiting times. The indicator is calculated by dividing the number of first visits guaranteed within the regional plan for governing waiting times (Piano regionale governo liste di attesa - PRGLA) timescales by the number of total first visits supplied to the residents of Tuscany. Again, data have been reported for each local health district.

### Statistical analysis

A high-low ratio of the total rate of first contact and follow-up cardiology visits during 2019–2021 has been run to address the first aim of this study. For each year, we divided the highest with the lowest rate of first contact and follow-up visits observed among Tuscany’s three local health authorities. Moreover, the same analysis has been applied to Tuscany’s 26 local health districts to analyse unwarranted variation among health districts. In addition to this analysis, we provided a bar chart to visualise the number of examinations that exceed or fall below the regional median rate of cardiology visits, divided per first contact, repeated first contact, and follow-up visits across Tuscan local health districts.

But what level of supply should be ensured to avoid a supply gap and thus ensure the delivery of the essential level of care? To identify a benchmark numerical value, the research team calculated the estimated annual visit rates required for healthcare providers in each local health district to meet the regional median. The districts belonging to the first quadrant in 2021 were selected. Given that the analysis was conducted in mid-2022, the reference year selected was 2021, from which the gap of volumes (V) to reach the median by the end 2022 was calculated:

$$\begin{array}{l}\:{V_{expected\:by\:end\:2022}} = \\\left( {{V_{2021}} + {V_{2021\:to\:reach\:median}}} \right) - \:{V_{recorded\:on\:1st\:semester\:2022}}\:\end{array}$$

Volumes were estimated based on the standardised rate per 1,000 residents (see Appendix A, Table A1).

Considering the non-existence of a standard rate, this quantitative tool to identify the quantitative effort required to reach the regional average of services provided per inhabitant, could support management and resource allocation decisions among managers and policymakers.

## Results

**Fig. 2:**
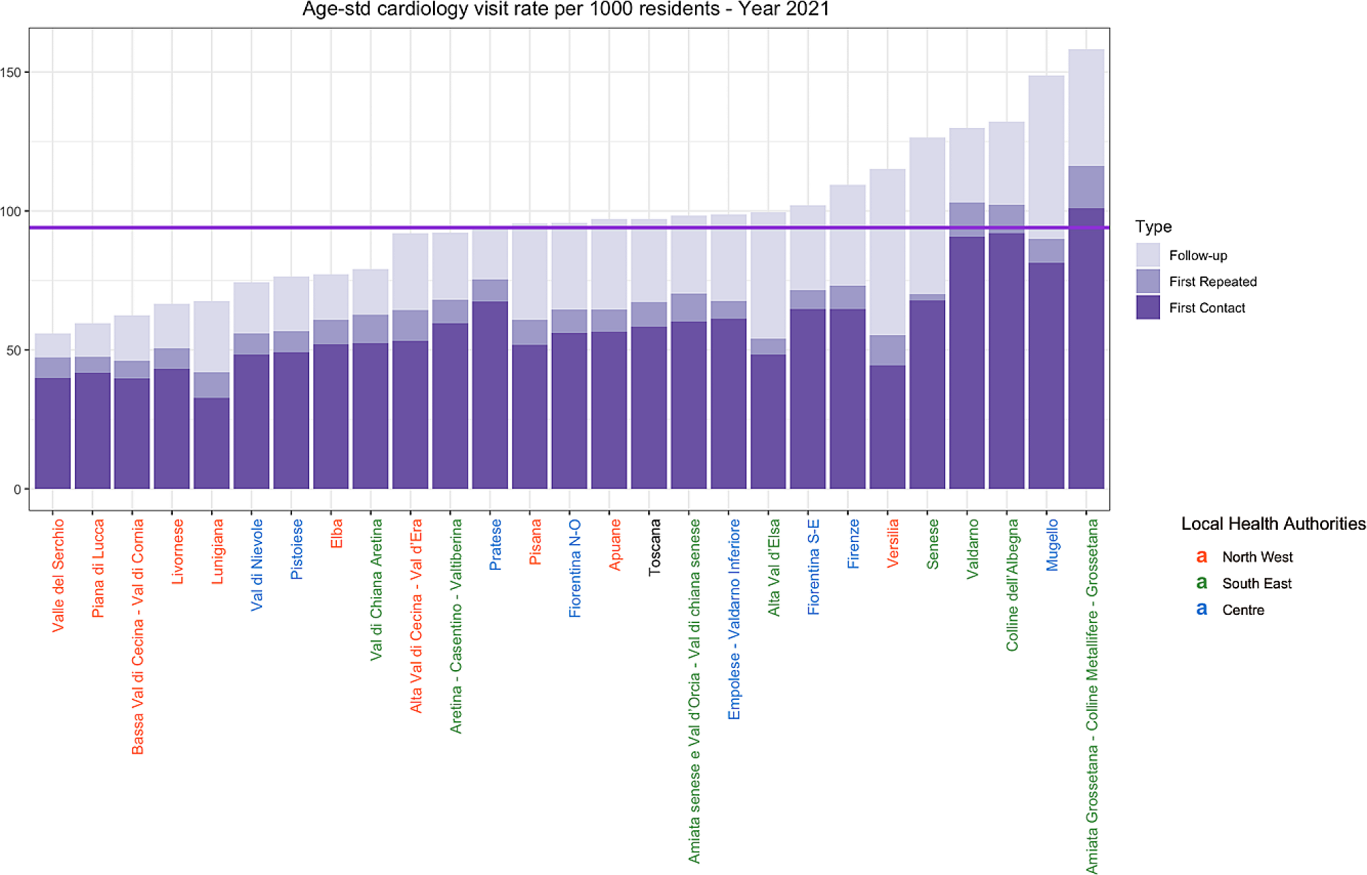
Bar chart illustrating the distribution of age-standardised cardiology visits rate per 1000 residents across local health districts of Tuscany, year 2021. Each colour represents one of Tuscany’s three local health authorities: red for Northwest LHA, green for Southeast LHA, and blue for Centre LHA. Visits are divided into first contact, first repeated, and follow-up visits. The purple horizontal line indicates the regional median value for the year 2021

In 2021, more than 400 thousand cardiology visits were provided in Tuscany, corresponding to an overall rate per 1000 residents of 97.04, with a regional median of 95.57. However, the distribution of cardiology visits rate across the 26 health districts of Tuscany showed a marked heterogeneity: the minimum value of 55.87 in Valle del Serchio is opposed to a maximum of 158.13 per 1000 residents in Amiata Grossetana – Colline Metallifere – Grossetana (Fig. 2), with an interquartile range of 28.95. This high variation among local health districts is also present for the years of 2019 and 2020. More precisely, an increasing trend can be found from 2019 to 2021, with a high-low ratio respectively of 2.10, 2.48, and 2.83. Moreover, during the same period, inter-local health authority variation – i.e., among Northwest, Southeast, and Centre – also showed an increasing tendency, with an increase of almost 31% of the high-low ratio (1.11 for 2019, 1.29 for 2020, and 1.45 for 2021). By analysing Fig. 2, most of the red- marked districts – all belonging to the sale LHS (North West) - have a low level of visit rates, illustrating that internal organisational models within the three LHAs are likely different.

In terms of types of visits, Fig. 2 illustrates the considerably higher number of first-contact visits compared to follow-up ones for the year 2021. Moreover, in all local health districts, repeated first-contact visits seem to be registered at a markedly high percentage (from 3 to 21% of the total amount of first-contact visits). These types of visits, which are follow-ups, have been prescribed and categorised inappropriately as first visits, which are ensured in a shorter time frame compared to follow-up appointments.

**Fig. 3:**
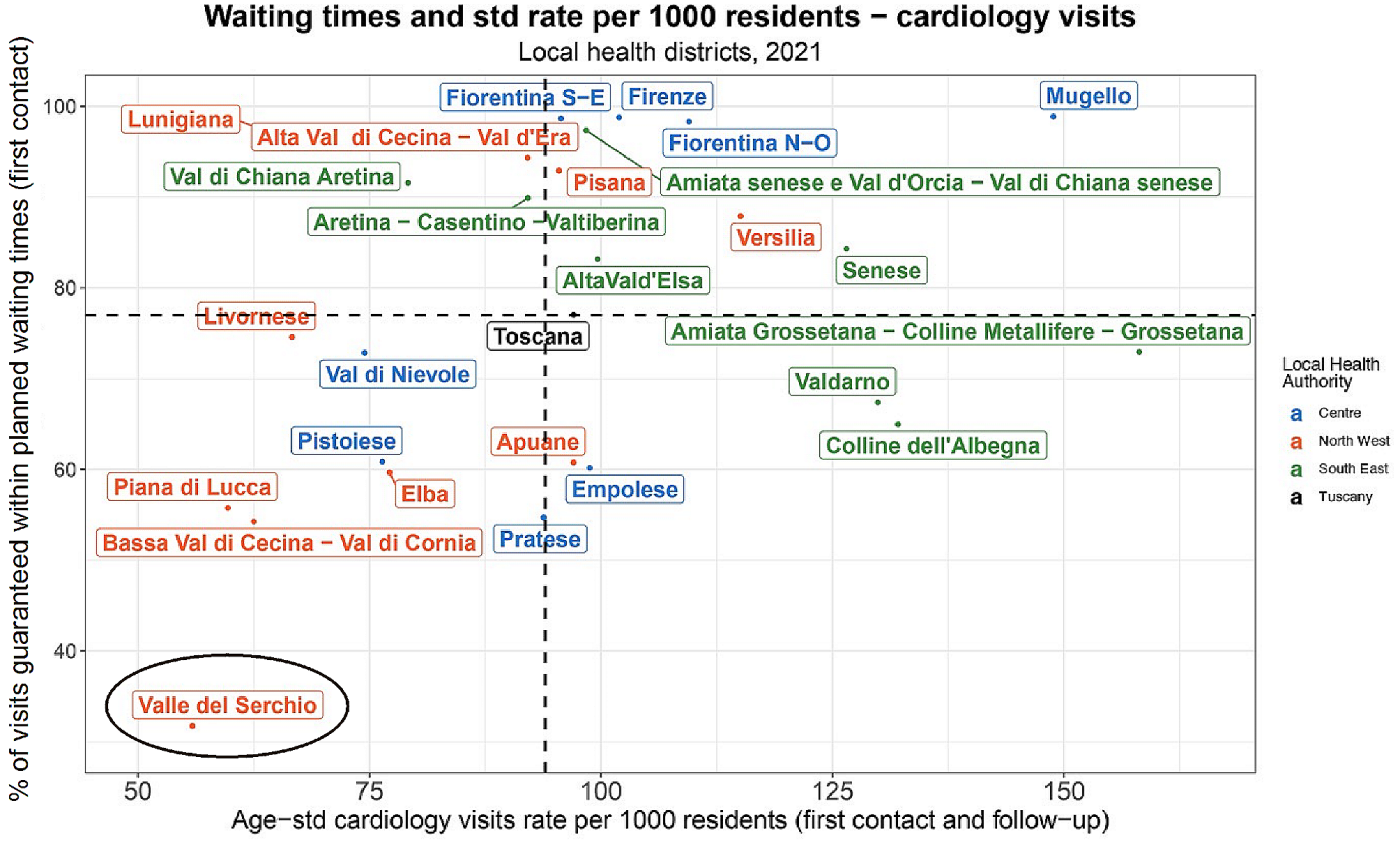
Matrix for waiting times and age-standardised cardiology visits rate per 1.000 residents, year 2021. Each colour represents one of Tuscany’s three local health authorities: red for Northwest LHA, green for Southeast LHA, and blue for Centre LHA. The median values of the moving average for the years 2019–2021 of the indicators define the quadrants

Analysing the two-dimensional matrix (Fig. 3), where the x-axis shows the cardiology visits rate per 1000 inhabitants, and the y-axis reports the percentage of visits delivered that respect waiting times, a lower rate of cardiology visits than the median value of the moving average of the years 2019–2021 is evident in seven out of ten local health districts belonging to the Northwest local health authority, in 2021. Contrariwise, considering the same indicator during the same period, six out of eight districts of the Southeast part of Tuscany are above the median. However, three of these health districts – i.e., Colline dell’Albenga, Valdarno, and Amiata Grossetana-Colline Metallifere – Grossetana – deliver a lower percentage of visits within the waiting times requested compared to the regional median. The Mugello rural health district, in the central part of Tuscany, scored the highest in the fourth quadrant, indicating the ability to manage many patients quickly, but with a possible hint of inappropriateness related to overprescription. Health districts belonging to the first quadrant represent the most critical situation. These areas experience a visit rate below the regional median, and furthermore, only a small percentage of these visits occur within the planned waiting times. More precisely, in 2021, among Tuscany’s health districts, the Valle del Serchio inner area scored the lowest rate of first contact and follow-up visits (55.87) and the lowest percentage of visits guaranteed within waiting times (31.73%).

The matrix of the year 2019 is shown in *Figure A.1* in Appendix A, demonstrating that differences in waiting times between districts were already present before Covid-19 and widened during the pandemic period. The pandemic had a negative overall impact on the capacity to deliver services, leading to increased waiting times across all district areas.

In September 2022, the 2021 matrix was presented and discussed in the presence of the Directorate General of Health and the LHAs’ top managers of the Region of Tuscany. Subsequently, the Directorate General of Health delineated specific objectives for each LHA, targeting the reduction of avoidable variation among health districts within a priority setting approach. Indeed, emphasis was placed on increasing visit volumes and reducing waiting times in districts falling within the first quadrant. More precisely, while efforts were urged across all quadrants, the primary focus was on increasing visit volumes in the critical first quadrant, given its potential impact on health outcomes due to the high risk of delivering a small number of visits relative to the patients’ needs and the long waiting times registered. Based on these directives, the authors provided the Region and each LHA with a quantitative estimate of the number of visits to be delivered for the districts belonging to the first critical quadrant, with the goal to achieve at least the regional median rate of visits provided. The LHAs’ managers analysed the situation in each district and put in place two different action strategies, considering the population dimension involved. As an example, in Tuscany’s Northwest LHA, in which districts belonging to the first quadrant were more than one, the types of interventions that the LHA put in place were different. Within the Northwest LHA, the Livornese district counts 170 thousand inhabitants [24], concentrated in the town area and suburbs. The absolute number of visits required to reach the regional median in 2022 (19,385 - Appendix A, Table A1) in this case was significant enough to justify the need to add a new cardiologist to enhance the provision of these services. A different situation occurred within the Valle del Serchio district, counting 53 thousand inhabitants and where the number of services to be delivered (6,338 – Appendix A, Table A1) was significantly lower and not enough to justify a new cardiologist in the area. Moreover, the Valle del Serchio district is an inner area, i.e. mountainous area with low population density, facing structural challenges associated with ageing and depopulation [25]. These areas experience a negative cycle marked by a migration of the younger population towards more urbanised centres, resulting in diminished attractiveness and employment opportunities, also within the healthcare sector, that contribute to overall impoverishment [25]. In these areas ensuring economic sustainability of health services is highly challenging due to the low population density and therefore, a broader strategy should be considered valuing interventions beyond traditional modalities.

**Fig. 4:**
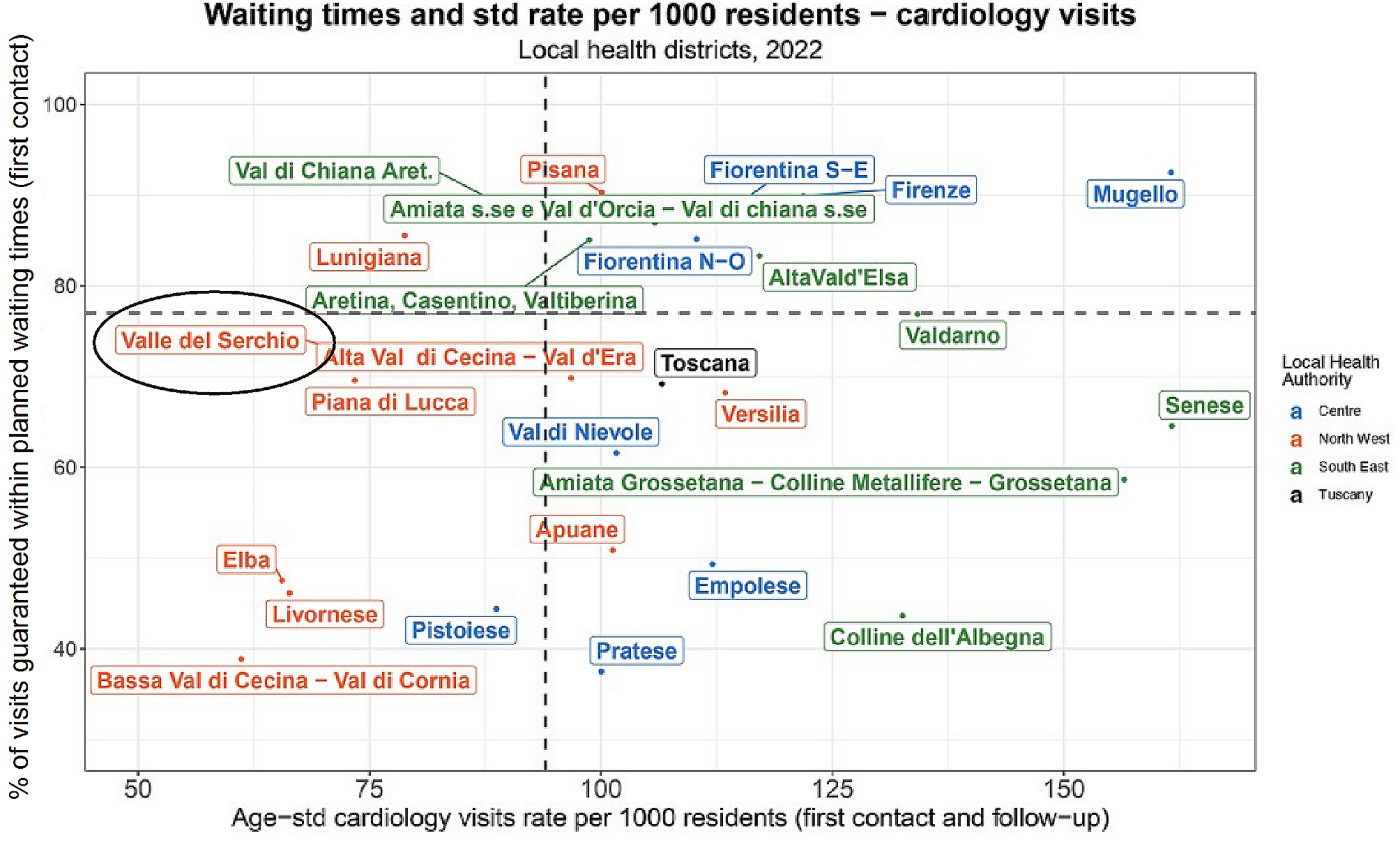
Matrix for waiting times and age-standardised cardiology visits rate per 1.000 residents, year 2022. Each colour represents one of Tuscany’s three local health authorities: red for Northwest LHA, green for Southeast LHA, and blue for Centre LHA. The median values of the moving average for the years 2019–2022 of the indicators define the quadrants.

It is within this specific context that the Pro-Heart project [26] was implemented in 2022 in the Valle del Serchio district. This initiative involved the Northwest LHA and the Gabriele Monasterio Foundation Hospital (Fondazione Gabriele Monasterio), a leading cardiology centre in Tuscany with hospital facilities in Massa and Pisa. This project was implemented with a dual purpose: firstly, thanks to the part-time presence of professionals of the Monasterio Foundation Hospital in the area, it enabled advanced training activities of local professionals. This not only gave the latter the opportunity to be trained and updated with the most recent clinical procedures, but also prevented them from feeling isolated. Secondly, the project aimed to meet the gap of demand for visits by integrating the presence of professionals alongside the adoption of new technologies, such as telemedicine. More specifically, efforts were made to train local healthcare professionals and nurses in adopting technologies that allow certain procedures and visits to be performed remotely i.e. holter monitor. In this regard, Fig. 4 illustrates how the implementation of the Pro-Heart project has yielded positive results within 2022: an increase in visits (from 3,705 to 4,601) and a reduction in waiting times (31.73% of visits were provided within planned waiting times in 2021, while in 2022 the percentage has risen to 73,35%).

From the experience gained from Pro-Heart, the intervention strategy was amplified within the Proximity Care^1^ project. Developed as a research project by the Sant’Anna School of Advanced Studies, in collaboration with the local municipality and the Local Health Authority, this initiative has received financial support from the “Fondazione Cassa di Risparmio” of Lucca. By integrating a range of multidisciplinary expertise, including clinical, managerial, telecommunications, and engineering, the aim has been to increase patient visits and establish a systematic approach to managing chronic patients. This has involved employing different technological tools and facilitating collaboration among all stakeholders, ultimately creating an improved model of patient care. Hence, in addition to strengthening the adoption of video-consultations, efforts have been made to create stronger connections among general practitioners (GPs) and other professionals. To do this, a shared data environment within a unified electronic clinical record has been put in place. Hence, the importance of establishing networks among specialists (also from remote), local healthcare professionals, and general practitioners (GPs) serving the area has been acknowledged and strengthened. Moreover, greater emphasis on prevention has been identified as key in this context [27]; the population in this area needs to take greater responsibility for their health by adopting healthier lifestyles, facilitated through peer-to-peer interactions among residents. This can be achieved through the creation of strong collaborations among third sector organisations, healthcare facilities, and citizens. As the project is currently ongoing, the upcoming years will be crucial for studying the quite different situation in the Livorno district, where it has been more complex for the LHA to increase the number of the available cardiologists and therefore to supply visits, leading to a sharp reduction of services delivered in time. Indeed, in 2022, the rate of visits further declined compared to the previous year (from 13,515 to 13,368 - see Appendix A, Table A1) and only 46.14% of visits were provided within the planned waiting times (74.58% in 2021).

## Discussion

The massive differences across health districts in terms of cardiology visit rates and waiting times, mark a profound heterogeneity in Tuscany, not due to patients-preference [28]. Although the principal focus of the study is intra-regional variation rather than pre- and post-pandemic variation, the presence of the Covid-19 pandemic is noteworthy as data collection coincides with this period. It is important to acknowledge the incidental influence of the Covid-19 pandemic on the data under analysis, as it doesn’t change the discussion on unwarranted geographic variation. As indicated by the high-low ratio from 2019 to 2021, high variations in resource input and utilisation of services were already evident before Covid-19, consistent with the Wennberg model [3, 6], and increased during 2020, when disparities acquired sharpness and escalated in the Covid-19 pandemic scenario [29]. The repercussions of the shock of 2020 on healthcare systems are, and probably will be, observed throughout the years to come. Adopting the matrix for cardiological visits has been a valuable tool for managers and policymakers to identify the areas where fewer visits were delivered and where reducing waiting times was an urgent issue (first quadrant). Each quadrant of the matrix proposed in this study suggests different interventions to reduce unwarranted variation. Districts that are in the right-side quadrants, where the number of visits delivered is strongly above the median, surely need a collaboration with GPs and specialists to reduce overprescription. However, with the perspective of reducing potential harm to patients and mitigating risks for future outcomes, greater emphasis should be placed on the first quadrant, where managers hold greater responsibility, as they oversee the resource allocation decision process. Indeed, while an excess of services can be detrimental to the health system’s sustainability, as it may result in a waste of resources, a significant shortage of supply of visits that some districts are facing in Q1, can profoundly affect the population’s health. This is especially crucial when linked to ambulatory care sensitive conditions (ACSCs). Limited access to healthcare, characterised by long waiting times and low visit rates, poses a serious threat to health outcomes. Delays in diagnosis and treatment can exacerbate existing conditions, increasing the risk of hospitalisation and even death [30–32]. This issue is particularly critical in inner areas, where underserved populations face even greater challenges in accessing timely care [33, 34]. This often translates to higher mortality rates and lower life expectancy among those living in remote locations [35], emphasising the urgent need for targeted strategies to improve healthcare access in these areas. Furthermore, while implementing initiatives to modify the clinicians’ prescription behaviours and establishing a sharing process with professionals to reduce overprescription requires time, interventions to increase supply in the first quadrant are more directly achievable. Such measures can be put in place by management authority, who possesses the ability to allocate and reallocate resources directly among districts.

While there is no established standard rate of outpatient cardiologic visits supported by scientific evidence, quantifying the volumes of visits to be delivered, in line with the regional median value, can serve as a proxy for managers and policymakers useful to fix the type of action to put in place and to identify more precisely what can constitute the “essential level of care” as foreseen by Italian law [36]. Furthermore, providing managers and policymakers with a quantitative estimate of the required number of visits to address shortages in certain districts, facilitates scaling the type of intervention. For large health authorities such as the Northwest LHA, encompassing over 1.2 million residents across a vast geographic area, this tool can be crucial in the resource allocation process and budget management among its 10 local health districts.

In the described cases, based on the results of 2021, the management of the Northwest LHA, guided by the quantitative assessment of volumes required to attain the median value, identified a need for additional cardiologists in the Livornese health district. However, in 2022, due to a shortage of healthcare professionals, this district was unable to achieve the goal of increasing cardiology visits necessary to reach the median target (see Appendix A, Table A1).

Conversely, an improvement in the rate of cardiology visits and waiting times can be observed within the Valle del Serchio district in 2022. Located in an inner area, where it is difficult to attract new and young specialists as they perceive these locations as unappealing for professional growth [37–39], the engagement of tertiary specialised hospitals in a perspective of hospital-territory collaboration played a crucial role. The implementation of the Pro-Heart project exemplifies this shift, as it emphasises the establishment of robust networks among a specialised hospital centre (Monasterio Hospital Centre) and local healthcare professionals.

Moreover, the project aimed to serve as the first experiment in implementing telemedicine activities in the Valle del Serchio district. Through initiatives focused on training local healthcare professionals and nurses in remote healthcare technologies, the project aimed to extend its reach beyond physical presence, thereby addressing geographical disparities in healthcare access. Therefore, by leveraging telemedicine and innovative technologies, it is possible to establish networks across considerable geographic distances, with highly specialised centres supporting the most remote geographic regions. This project has evidenced the need and challenge for large and/or specialised hospitals, in acquiring knowledge and tools to enable the growth of telemedicine, hence guaranteeing support to hospitals and the population of remote areas.

Within the realm of telemedicine, the Proximity Care project is currently experimenting with creating stronger networks not only between specialised hospital centres, such as the Monasterio Foundation Hospital, and local specialists, but also with local GPs, by implementing a shared electronic clinical record. Here, all professionals can share patients’ data, with the aim of creating an integrated environment that ultimately benefits health outcomes. Overall, this approach has yielded significant satisfaction among GPs in this district, fostering a sense of reduced isolation and increased participation in the context of scientific and clinical innovation.

The study has limitations that represent a crucial starting point for further research. First, the potentially different prevalence of cardiovascular diseases across different geographical areas, which could affect visit rates. In 2021, the age-standardised rate of chronic heart failure patients per 1000 residents [40] varied from 15.06 to 23.49 among the local health districts of Tuscany. While this aspect certainly needs further examination, it is unlikely that prevalence alone justifies a high-low ratio of 2.83 in 2021, which is mostly explained by organisational factors.

Another limitation is related to the setting: the study is limited to Tuscany, one of the twenty Italian regions. This limitation represents a further research opportunity as the proposed tools for supporting policy measures and management practices can be extended to other regions and disciplines within outpatient settings. Indeed, after having tested the strategies proposed in this paper in the Valle del Serchio health district, other projects are being implemented within other inner areas.

## Conclusion

This study explores the critical issue of guaranteeing equitable access in outpatient cardiovascular services, presenting the case of the region of Tuscany, Italy. The interconnected challenges of geographical variation and waiting times have been explored through the lens of a comprehensive matrix developed by Nuti and Vainieri [5]. The findings highlight the vulnerability of certain health districts, revealing a significant heterogeneity in service provision, even more pronounced during the challenges posed by the Covid-19 pandemic. Based on these premises, the matrix serves as a valuable tool for policymakers and managers, enabling them to identify areas with lower service delivery and urgent issues related to waiting times compared to neighbouring areas. The computation of volumes of visits needed to reach regional averages provides a quantitative baseline, offering essential insights for efficient resource allocation, strategic planning and magnitude of intervention.

The study identifies districts in the first quadrant of the matrix as priority intervention areas. It underscores the necessity of deploying different strategies tailored to the specific challenges of each district, taking into account factors such as population density and territorial characteristics. In particular, inner areas such as the Valle del Serchio district, face structural challenges necessitating innovative strategies, like the ones proposed by Pro-Heart and Proximity Care projects, which have been presented in this study as an example of novel approaches that could be pursued at larger scale for similar rural areas at national or international level. These projects exemplify the importance of adopting innovative technologies and emphasise the importance of prevention in those areas where direct management of supply might be difficult. Hence, a paradigm shift is needed: organisational models and technologies can be designed and experimented in geographic regions that might benefit from these the most, such as inner areas.

In the face of these challenges, the study contributes to the ongoing discourse on unwarranted variation. It underscores the importance of tailored interventions by illustrating examples of projects that have been implemented to increase visit rates, acknowledging the diversity of healthcare challenges across geographical areas. As healthcare systems worldwide face evolving complexities, the study’s findings and tools can guide future research, policymakers and managers toward achieving more equitable, efficient, and responsive healthcare services.

## Electronic supplementary material

Below is the link to the electronic supplementary material.

Supplementary Material 1

## Data Availability

Data mentioned in the manuscript are freely available on the internet (https://performance.santannapisa.it/pes/start/start.php).

## Footnotes

1 https://www.proximitycare.it.
