# Supplementary material for: Setting priorities in outpatient cardiovascular care to guarantee equitable access: the case of Tuscany region
Source: Res Health Serv Reg. 2024 Aug 7;3:11. doi: 10.1007/s43999-024-00047-9 (PMC11802940; doi:10.1007/s43999-024-00047-9)
Supplement: Supplementary file 1 — Supplementary Material 1 [file 43999_2024_47_MOESM1_ESM.docx]

**Appendix A**


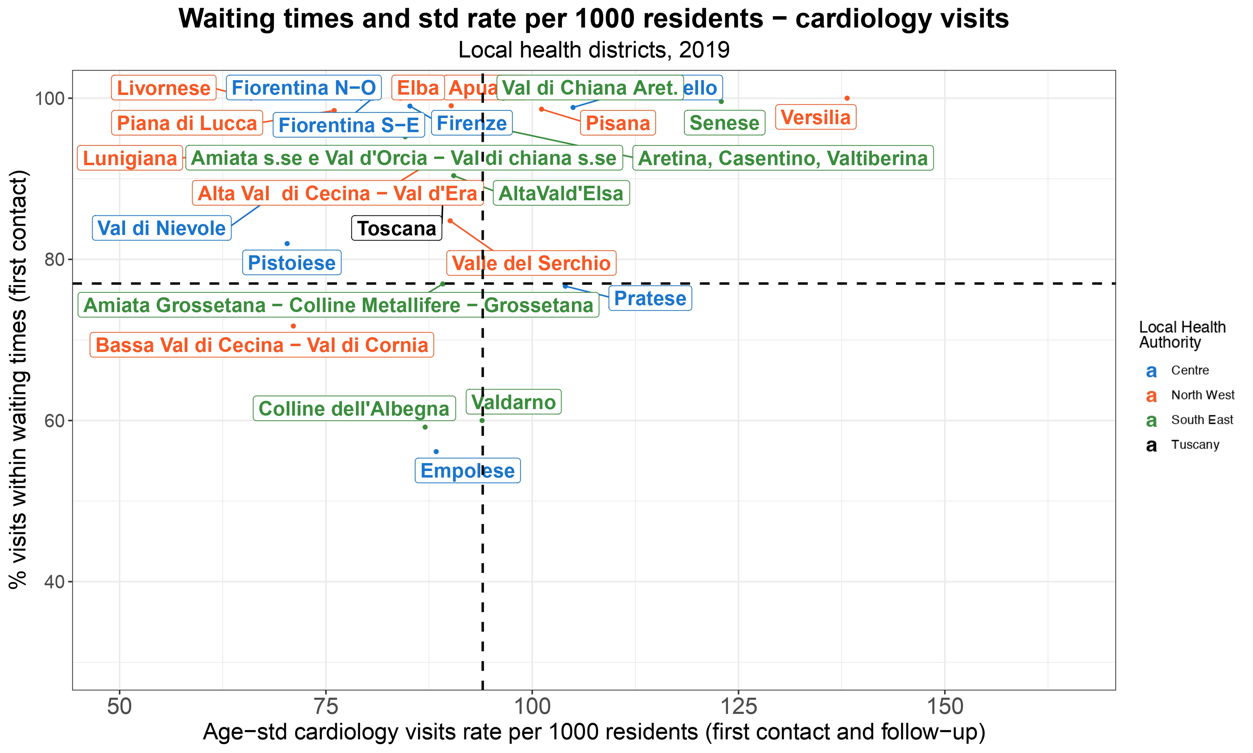


***Figure A1*.** *Matrix for waiting times and age-standardised cardiology visits rate per 1.000 residents, year 2019. Each colour represents one of Tuscany’s three local health authorities: red for Northwest LHA, green for Southeast LHA, and blue for Centre LHA. The median values of the moving average for the years 2019-2021 of the indicators define the quadrants.*

| **Local health districts** | **Volumes 2021 (first visits and follow-ups)** | **Age-std rate per 1000 residents - 2021** | **Target age-std rate per 1000 residents for 2022 (=regional median 2021)** | **Total annual target volumes for 2022 based on 2021** | **Total volumes provided in 2022** | **Target volumes for 2022 - volumes provided in 2022** | **Percentage achievement of the target** |
| --- | --- | --- | --- | --- | --- | --- | --- |
| Bassa Val di Cecina - Val di Cornia | 10,458 | 62.49 | 95.57 | 15,993 | 10,190 | -5,803 | -179% |
| Elba | 2,858 | 77.17 | 95.57 | 3,539 | 2,449 | -1,090 | -117% |
| Livornese | 13,515 | 66.63 | 95.57 | 19,385 | 13,368 | -6,017 | -135% |
| Piana di Lucca | 11,215 | 59.70 | 95.57 | 17,954 | 13,897 | -4,057 | -102% |
| Valle del Serchio | 3,705 | 55.87 | 95.57 | 6,338 | 4,601 | -1.737 | -121% |
| Pistoiese | 15,118 | 76.38 | 95.57 | 18,918 | 17,378 | -1,540 | -24% |
| Val di Nievole | 9,983 | 74.47 | 95.57 | 12,812 | 13,535 | 723 | 4% |

***Table A1.*** *Computation of cardiology visits of 2022 (first visits and follow-ups) needed to reach the regional median. Only local health districts belonging to the first quadrant of the matrix for the year 2021 have been considered.*
